# Case Report: Double Germline Mutations in *BRCA1* and *MSH2* in a Patient With Mixed Serous-Endometrioid Endometrial Carcinoma

**DOI:** 10.3389/fmed.2020.581982

**Published:** 2020-11-03

**Authors:** Hong Zheng, Mingming Yuan, Huanwen Wu, Rongrong Chen, Yunong Gao

**Affiliations:** ^1^Key Laboratory of Carcinogenesis and Translational Research (Ministry of Education), Department of Gynecology, Peking University Cancer Hospital & Institute, Beijing, China; ^2^Geneplus-Beijing, Beijing, China; ^3^Department of Pathology, Molecular Pathology Research Center, Peking Union Medical College Hospital, Chinese Academy of Medical Sciences and Peking Union Medical College, Beijing, China

**Keywords:** double germline mutations, mixed serous-endometrioid endometrial carcinoma, next-generation sequencing, tislelizumab, tumor heterogeneity

## Abstract

Mixed serous-endometrioid endometrial carcinoma is a type of endometrial cancer with relatively low incidence. The genetic factors contributing to the tumorigenesis of mixed carcinoma remains to be explored. Here, we report the first identification of two germline mutations in *BRCA1* and *MSH2* in a woman with mixed serous papillary adenocarcinoma and endometrioid carcinoma. Immunohistochemistry analysis showed loss of MSH2 and MSH6 protein expression in the endometrioid component. The patient showed partial response to tislelizumab treatment following progression on chemotherapy. Two germline mutations in *BRCA1* and *MSH2* may collectively promote the tumorigenesis of uterine endometrium with two distinct histological components.

## Introduction

Endometrial carcinoma (EC) is the second most common gynecologic malignancy in China, with an estimated 63,400 new cases and 21,800 deaths in 2015 (1). The vast majority of ECs are sporadic, and hereditary tumor syndrome [most commonly Lynch syndrome (LS)] accounts for ~5% of cases (2). LS is characterized by the identification of germline pathogenetic mutations in mismatch repair (MMR) genes (mainly including *MLH1, MSH2, MSH6, PMS2*), microsatellite instability and loss of MMR protein expression, which are usually related to endometrioid histology. Besides, Shu et al. reported that *BRCA1* germline mutations may increase the risk for serous or serous-like ECs (3). Concurrent pathogenic variants in different genes in one individual is extremely rare. In this study, we identified two germline pathogenic mutations in the *BRCA1* and *MSH2* genes in a patient with mixed endometrioid and serous EC (EEC-SC).

## Case Presentation

**Figure 1:**
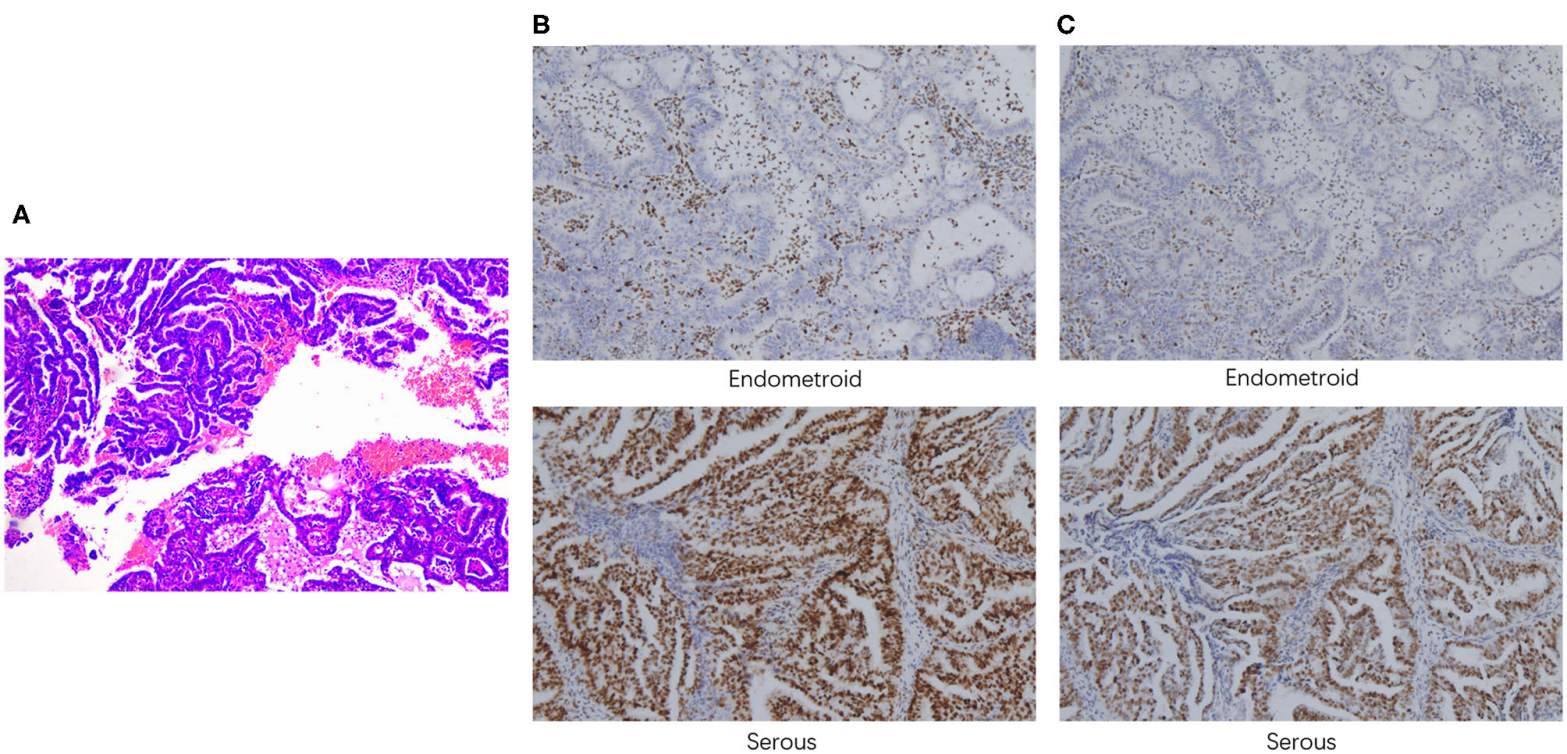
Pathological findings. **(A)** H&E staining demonstrated the presence of both serous and endometrioid components. Immunohistochemistry staining of MSH2 **(B)** and MSH6 **(C)** (100x magnification).

A 52-year-old woman underwent laparoscopic total hysterectomy, bilateral salpingo-oophorectomy, omentectomy, and dissection of pelvic and para-aortic lymph nodes in June, 2018. Pathological findings confirmed the diagnosis of stage IIIA serous papillary adenocarcinoma mixed with endometrioid carcinoma with squamous differentiation (~70% for serous carcinoma component and 30% for endometroid carcinoma component, respectively) (Figure 1A), with tumor metastasis to both fallopian tubes. Immunohistochemistry analysis showed loss of MSH2 (Figure 1B) and MSH6 (Figure 1C) protein expression in the EEC component. She received 6 cycles of paclitaxel and carboplatin as adjuvant therapy with complete response. Postoperative routine follow-up examination in May, 2019 showed that serum CA125 was elevated (71.66 U/ml), but CT examination did not reveal any abnormalities. The level of serum CA125 increased to 100.90 U/ml after 1 month, and ultrasound examination also showed enlarged paraaortic lymph node of 2.5 cm in diameter, suggesting tumor recurrence. Rechallenge of paclitaxel and carboplatin for one cycle failed with continued increase of CA125 to 120.1 U/ml. Subsequently, the patient switched to oxaliplatin combined with pegylated liposomal doxorubicin for one cycle with primary progression. Evaluation after the chemotherapy on August 1, 2019 showed serum CA125 level increased to 231.6 U/ml. CT scan suggested an enlarged left para-aortic lymph node (37^*^30 mm) (Supplementary Figures 1A,E).

**Figure 2:**
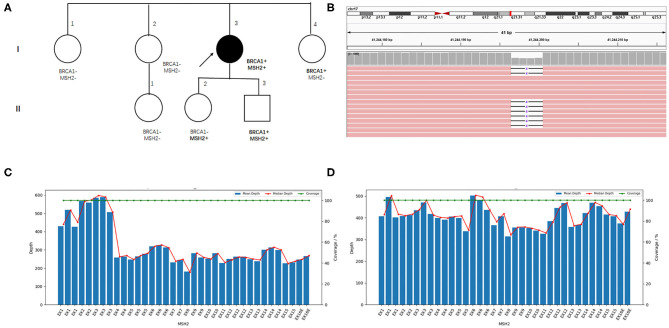
Family pedigree and germline mutations identified in the index patient. **(A)** Family pedigree. The index patient is indicated with an arrow, and the circle filled with black color denotes endometrial cancer. **(B)** Visualized sequencing data shows the *BRCA1* variant. *MSH2* exons coverage depth analysis in the index patient **(C)** and another sample with the same batch **(D)**.

The patient came to our hospital for second opinion. To seek for potential targeted therapies and immunotherapies, paired tumor-normal next-generation sequencing of 1,021 cancer-related genes was performed using tumor tissue and peripheral blood. Of great interest, two heterozygous germline mutations in *BRCA1* (NM_007294.3 c.3348_3351delAGTT p.V1117Rfs^*^11) and *MSH2* (large deletion of exons 4-16) were identified (Figures 2B–D). Besides, a total of 72 somatic mutations were detected, including putative or known functional mutations in *PTEN, ARID1A, TP53, FBXW7*, and *KRAS* (Supplementary Table 1). In addition, genetic testing results showed that microsatellites were highly unstable, and tumor mutation burden was extremely high (51.84 muts/Mb).

To access the cancer risk for the family members of the patient, Sanger sequencing and RT-PCR were performed to confirm the presence of germline *BRCA1* and *MSH2* mutations in her family members, although she has no family history of cancer. Her younger sister (I-4) and daughter (II-2) carry the *BRCA1* and *MSH2* mutations, respectively. Unfortunately, her son harbors both the *BRCA1* and *MSH2* mutations (Figures 2A, Supplementary Figures 2, 3).

The patient was then treated with 200 mg intravenous tislelizumab every 3 weeks from September 4, 2019. Partial remission was achieved at 9 weeks after treatment with the shrinkage of an enlarged left para-aortic lymph node (Supplementary Figures 1B,F). In addition, the normalization of serum CA125 (12.28 U/ml) was observed after 2 months of treatment. CT scan at 15 weeks after treatment demonstrated continuous shrinkage of the enlarged lymph node (Supplementary Figures 1C,G). She has been on treatment for 4 months, and discontinued the treatment for 2 months due to the impact of COVID-19 outbreak. Fortunately, the CT scan did not reveal progression as a result of drug interruption (Supplementary Figures 1D,H). She continued the treatment and is still in follow-up. During the treatment, she experienced grade 1 treatment-related elevation of alanine transaminase (ALT) and aspartate transaminase (AST), which was relieved after symptomatic treatment.

## Discussion

The case illustrates that the genetic factors of ECs are complex and may result in different histologic presentations. *MSH2* large deletions were identified in 6.4% (28/439) families with LS (4). In this case, a novel heterozygous *MSH2* large deletion was identified in the index patient using a well-designed panel-based NGS test. This reminds us that professionals for genetic variants interpretation should be aware of this rare type of mutation in practice. The relationship between mutations in the *BRCA1* gene and EC is controversial. Multiple studies found that *BRCA* mutation carriers may have an elevated risk of EC, while others suggested that the increased risk may be associated with tamoxifen treatment (3, 5–7). In our case, the index patient did not have history of breast cancer and tamoxifen treatment. A large retrospective study showed that the incidence of serous/serous-like ECs in *BRCA1* mutation carriers is significantly higher than expected. Biron-Shental et al. also found that high rate of *BRCA* germline mutation in SC patients accompanied by strong familial cancer history may indicate that SC is a part of HBOC (8). Therefore, we speculated that the SC component in the patient may be associated with the *BRCA1* germline mutation. Two germline mutations in *BRCA1* and *MSH2* may collectively promote the tumorigenesis of a single lesion with two distinct pathological components.

EC can be routinely classified into two distinct histological subtypes. Type I (~80–90%, mainly endometrioid adenocarcinoma) and type II (relatively uncommon, primarily serous and clear cell adenocarcinoma) tumors are distinct at the molecular level. High frequency of *POLE, PTEN, CTNNB1, PIK3R1, ARID1A, KRAS* mutations and microsatellite instability are found in type I tumors, whereas mutations in *TP53* and *FBXW7*, and somatic copy number alterations are more frequently found in type II carcinomas (9, 10). Coenegrachts et al. found that in majority of the cases, SC and EEC components in mixed EEC-SC exhibit distinct molecular characteristics, but have similar mutation profiles compared to SC and EEC cancers, respectively (11, 12). In the present case with mixed histological components, frequently mutated genes in endometrioid tumors (*PTEN, ARID1A, KRAS*) and serous tumors (*TP53, FBXW7*) are all mutated. Our results were consistent with their findings and supported the divergent clonal evolution in mixed ECs.

Immune checkpoint inhibitors provide an optional treatment strategy for patients with LS-related EC. In 2017, pembrolizumab, a mono-clonal antibody targeting programmed death receptor-1 (PD-1), was approved for microsatellite instability–high (MSI-H)/mismatch-repair–deficient (dMMR) solid tumors that have progressed after prior therapy and have no satisfactory alternative treatment options. A phase II study of pembrolizumab monotherapy in patients with MSI-H/dMMR endometrial cancer (*n* = 49) demonstrated an objective response rate (ORR) of 57.1% (95% CI, 42.2–71.2%), with a median progression-free survival (PFS) of 25.7 months (95% CI, 4.9 months to not reached) (13, 14). Tislelizumab, another anti PD-1 antibody, has been approved by NMPA for the treatment of recurrent and refractory classical Hodgkin lymphoma, as well as previously treated locally advanced or metastatic urothelial carcinoma with PD-L1 high expression. Multiple clinical studies demonstrated that tislelizumab monotherapy was well tolerated and effective in patients with advanced solid tumors, including urothelial, lung and gastric carcinoma, with the objective response rate ranging from 13 to 25% (15–17). To date, no clinical trial has been conducted to investigate the clinical activity of tislelizumab in patients with endometrial cancer. In our index patient, microsatellites were highly unstable. She has a germline mutation in *MSH2*, while IHC showed MSH2 and MSH6 expression were lost in the EEC component. She received tislelizumab with a good response after progression on multiple lines of chemotherapy. Due to the identification of the *BRCA1* germline mutation, poly ADP-ribose polymerase inhibitors monotherapy or combined with immunotherapy may be used in the subsequent lines of treatment. Tumors with *BRCA1/2* pathogenic mutations have higher level of genomic instability, and this may generate more neoantigens, which may be associated with better efficacy when receiving treatment with immune checkpoint inhibitors (18). Based on this rational, combined treatment with PARP inhibitors and immunotherapies has shown promising efficacy in multiple clinical trials (19, 20). The combination of tislelizumab with a novel PARP inhibitor—pamiparib was evaluated in solid tumors in a phase Ia/b clinical trial. Ten (20%) of 49 patients achieved an objective response, including two complete responses and eight partial responses (21).

In conclusion, this is the first report of two germline mutations in *BRCA1* and *MSH2* identified in a woman with mixed EEC-SC. Tumor heterogeneity at the level of germline and somatic aberrations may collectively promote the histological divergence in mixed EEC-SC.

## Data Availability Statement

The original contributions presented in the study are included in the article/Supplementary Materials, further inquiries can be directed to the corresponding author/s.

## Ethics Statement

The studies involving human participants were reviewed and approved by ethics committee of Peking University Cancer Hospital & Institute. The patients/participants provided their written informed consent to participate in this study. Written informed consent was obtained from the individual(s) for the publication of any potentially identifiable images or data included in this article.

## Author Contributions

YG: conception and design and study supervision. HW and RC: acquisition of data. HZ and MY: analysis and interpretation of data and writing, review, and/or revision of the manuscript. All authors contributed to the article and approved the submitted version.

## Conflict of Interest

MY and RC were employed by the company Geneplus-Beijing. The remaining authors declare that the research was conducted in the absence of any commercial or financial relationships that could be construed as a potential conflict of interest.

## References

[1] Chen W Zheng R Baade PD Zhang S Zeng H Bray F Cancer statistics in China, 2015. CA Cancer J Clin. (2016) 66:115–32. 10.3322/caac.21338 26808342

[2] Urick ME Bell DW. Clinical actionability of molecular targets in endometrial cancer. Nat Rev Cancer. (2019) 19:510–21. 10.1038/s41568-019-0177-x 31388127 PMC7446243

[3] Shu CA Pike MC Jotwani AR Friebel TM Soslow RA Levine DA. Uterine cancer after risk-reducing salpingo-oophorectomy without hysterectomy in women with BRCA mutations. JAMA Oncol. (2016) 2:1434–40. 10.1001/jamaoncol.2016.1820 27367496 PMC5594920

[4] van der Klift H Wijnen J Wagner A Verkuilen P Tops C Otway R. Molecular characterization of the spectrum of genomic deletions in the mismatch repair genes MSH2, MLH1, MSH6, and PMS2 responsible for hereditary nonpolyposis colorectal cancer (HNPCC). Genes Chromosomes Cancer. (2005) 44:123–38. 10.1002/gcc.20219 15942939

[5] Thompson D Easton DF Breast Cancer Linkage Consortium. Cancer incidence in BRCA1 mutation carriers. J Natl Cancer Inst. (2002) 94:1358–65. 10.1093/jnci/94.18.1358 12237281

[6] Beiner ME Finch A Rosen B Lubinski J Moller P Ghadirian P. The risk of endometrial cancer in women with BRCA1 and BRCA2 mutations. A prospective study. Gynecol Oncol. (2007) 104:7–10. 10.1016/j.ygyno.2006.08.004 16962648

[7] Segev Y Iqbal J Lubinski J Gronwald J Lynch HT Moller P. The incidence of endometrial cancer in women with BRCA1 and BRCA2 mutations: an international prospective cohort study. Gynecol Oncol. (2013) 130:127–31. 10.1016/j.ygyno.2013.03.027 23562522

[8] Biron-Shental T Drucker L Altaras M Bernheim J Fishman A. High incidence of BRCA1-2 germline mutations, previous breast cancer and familial cancer history in Jewish patients with uterine serous papillary carcinoma. Eur J Surg Oncol. (2006) 32:1097–100. 10.1016/j.ejso.2006.03.032 16650962

[9] Kandoth C Schultz N Cherniack AD Akbani R Liu Y Shen H. Integrated genomic characterization of endometrial carcinoma. Nature. (2013) 497:67–73. 10.1038/nature12113 23636398 PMC3704730

[10] Lax SF. Pathology of endometrial carcinoma. Adv Exp Med Biol. (2017) 943:75–96. 10.1007/978-3-319-43139-0_3 27910065

[11] Coenegrachts L Garcia-Dios DA Depreeuw J Santacana M Gatius S Zikan M Mutation profile and clinical outcome of mixed endometrioid-serous endometrial carcinomas are different from that of pure endometrioid or serous carcinomas. Virchows Arch. (2015) 466:415–22. 10.1007/s00428-015-1728-5 25677978

[12] Gatius S Cuevas D Fernandez C Roman-Canal B Adamoli V Piulats JM. Tumor heterogeneity in endometrial carcinoma: practical consequences. Pathobiology. (2018) 85:35–40. 10.1159/000475529 28614814

[13] O'Malley D Marabelle A De Jesus-Acosta A Piha-Paul SA Arkhipov A Longo F 1044P - Pembrolizumab in patients with MSI-H advanced endometrial cancer from the KEYNOTE-158 study. Ann Oncol. (2019) 30:v425–6. 10.1093/annonc/mdz250.052

[14] Marabelle A Le DT Ascierto PA Di Giacomo AM De Jesus-Acosta A Delord J-P. Efficacy of pembrolizumab in patients with noncolorectal high microsatellite instability/mismatch repair-deficient cancer: results from the phase II KEYNOTE-158 study. J Clin Oncol. (2020) 38:1–10. 10.1200/JCO.19.02105 31682550 PMC8184060

[15] Deva S Lee J Lin C Yen C Millward M Chao Y 70O A phase Ia/Ib trial of tislelizumab, an anti-PD-1 antibody (ab), in patients (pts) with advanced solid tumors. Ann Oncol. (2018) 29(Suppl._10):mdy487.042 10.1093/annonc/mdy487.042

[16] BeiGene BeiGene Announces Clinical Results on Tislelizumab Presented at the 22nd Annual Meeting of the Chinese Society of Clinical Oncology (CSCO). BeiGene (2019).

[17] Ye D Liu J Zhou A Zou Q Li H Fu C First report of efficacy and safety from a phase II trial of tislelizumab, an anti-PD-1 antibody, for the treatment of PD-L1+ locally advanced or metastatic urothelial carcinoma (UC) in Asian patients. Ann Oncol. (2019) 30:v367 10.1093/annonc/mdz249.019

[18] Strickland KC Howitt BE Shukla SA Rodig S Ritterhouse LL Liu JF. Association and prognostic significance of BRCA1/2-mutation status with neoantigen load, number of tumor-infiltrating lymphocytes and expression of PD-1/PD-L1 in high grade serous ovarian cancer. Oncotarget. (2016) 7:13587–98. 10.18632/oncotarget.7277 26871470 PMC4924663

[19] Konstantinopoulos PA Waggoner SE Vidal GA Mita MM Fleming GF Holloway RW TOPACIO/Keynote-162 (NCT02657889): A phase 1/2 study of niraparib + pembrolizumab in patients (pts) with advanced triple-negative breast cancer or recurrent ovarian cancer (ROC)—Results from ROC cohort. J Clin Oncol. (2018) 36(15_Suppl.):106 10.1200/JCO.2018.36.15_suppl.106

[20] Bang Y-J Kaufman B Geva R Stemmer SM Hong S-H Lee J-S An open-label, phase II basket study of olaparib and durvalumab (MEDIOLA): results in patients with relapsed gastric cancer. J Clin Oncol. (2019) 37(4_Suppl.):140 10.1200/JCO.2019.37.4_suppl.140

[21] Friedlander M Meniawy T Markman B Mileshkin L Harnett P Millward M. Pamiparib in combination with tislelizumab in patients with advanced solid tumours: results from the dose-escalation stage of a multicentre, open-label, phase 1a/b trial. Lancet Oncol. (2019) 20:1306–15. 10.1016/S1470-2045(19)30396-1 31378459

