# Supplementary material for: Case Report: Double Germline Mutations in BRCA1 and MSH2 in a Patient With Mixed Serous-Endometrioid Endometrial Carcinoma
Source: Front Med (Lausanne). 2020 Nov 3;7:581982. doi: 10.3389/fmed.2020.581982 (PMC7670051; doi:10.3389/fmed.2020.581982)

Figure S1. CT scan with coronal and transverse views demonstrated the continuous shrinkage of the enlarged left para-aortic lymph node during the period of tislelizumab treatment. The circles mark the location of the tumor. (A) and (E) represent the enlarged left para-aortic lymph node before tislelizumab treatment, (B) and (F) at 9 weeks after treatment, (C) and (G) at 15 weeks after treatment, (D) and (H) at 28 weeks after treatment.

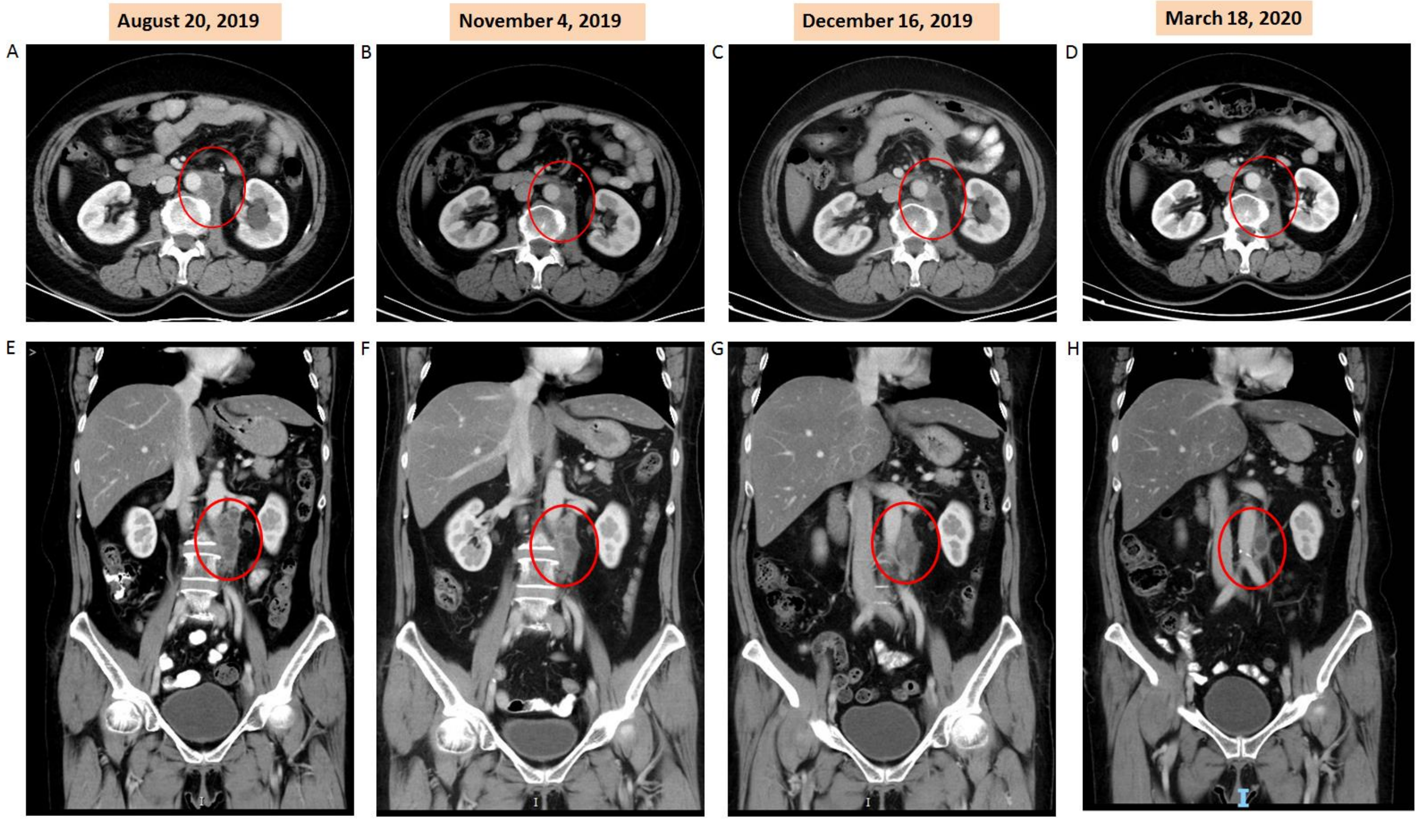

Figure S2. Sanger sequencing of the *BRCA1* c.3348_3351delAGTT variant in the family shows that the proband (Ⅰ-3), her younger sister (Ⅰ-4) and her son (Ⅱ-3) were all mutation carriers. Red arrow indicates the location of the mutation.

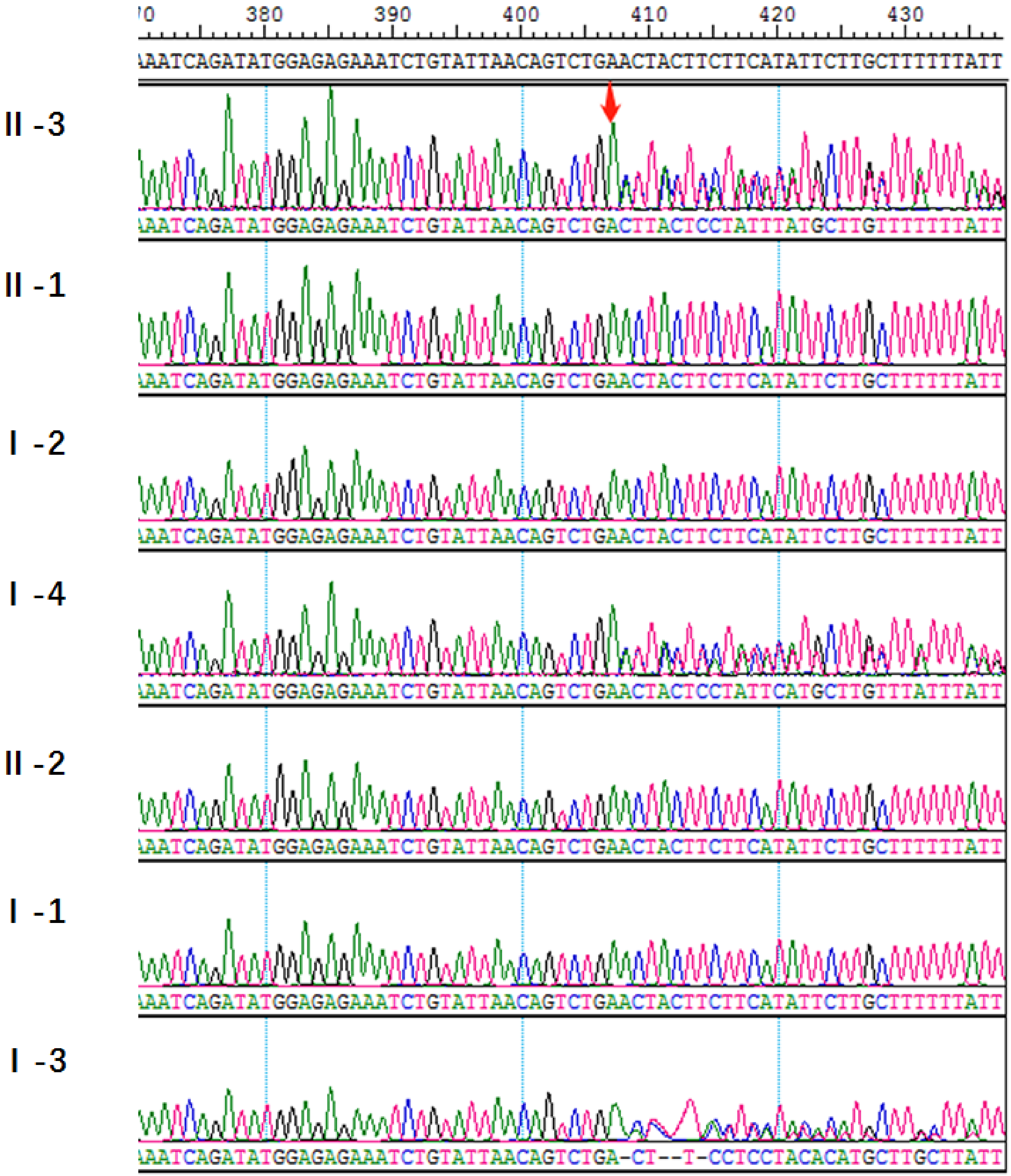

Figure S3. Relative fold change in gene expression of *MSH2* assessed by RT-PCR. The relative expression was determined using the 2^-△△Ct method and was normalized to the *ATCB* gene and normal control sample. A ratio of 1±0.2 indicates a normal diploidy, and a ratio of 0.5±0.2 indicates a deletion. The figure shows that Ⅰ-3, Ⅱ-2 and Ⅱ-3 carried heterozygous deletion in exons 4-16 of the *MSH2* gene.

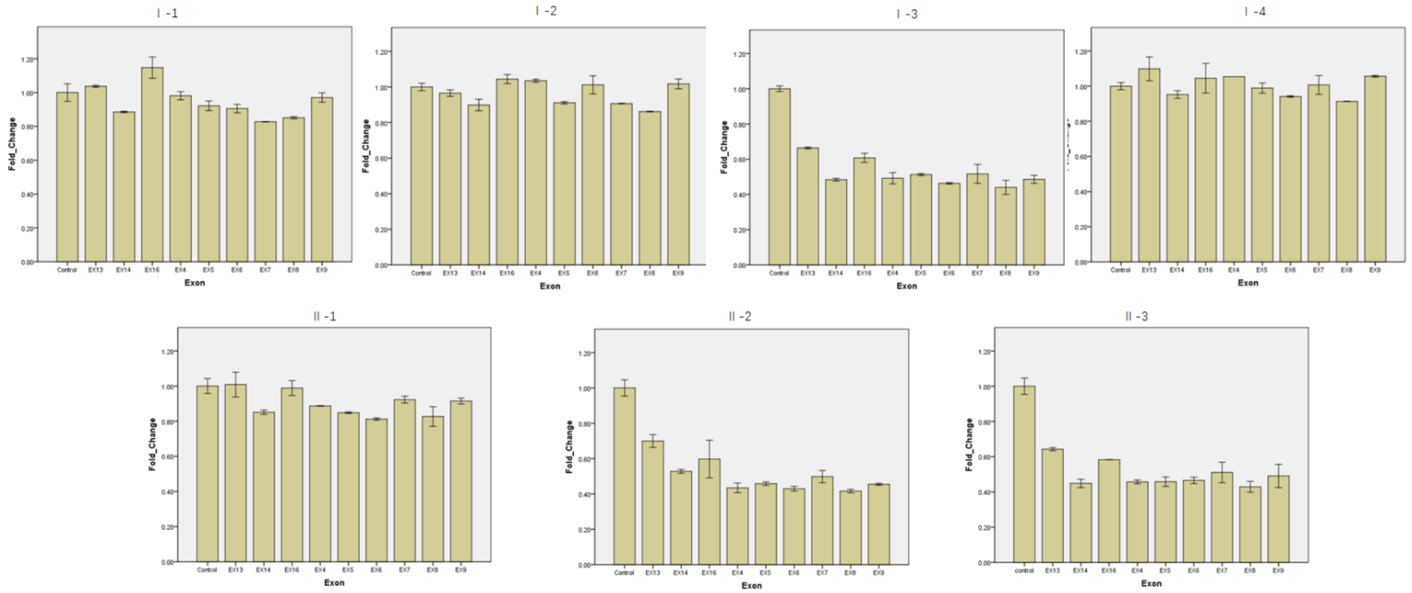

Supplement: Supplementary file 1 [file Data_Sheet_1.PDF]
